# AMPK-Dependent Metabolic Regulation by PPAR Agonists

**DOI:** 10.1155/2010/549101

**Published:** 2010-08-08

**Authors:** Woo Hyung Lee, Sang Geon Kim

**Affiliations:** College of Pharmacy and Research Institute of Pharmaceutical Sciences, Seoul National University, Seoul 151-742, Republic of Korea

## Abstract

Comprehensive studies support the notion that the peroxisome proliferator-activated receptors, (PPARs), PPAR*α*, PPAR*β*/*δ*, and PPAR*γ*, regulate cell growth, morphogenesis, differentiation, and homeostasis. Agonists of each PPAR subtype exert their effects similarly or distinctly in different tissues such as liver, muscle, fat, and vessels. It is noteworthy that PPAR*α* or PPAR*γ* agonists have pharmacological effects by modulating the activity of AMPK, which is a key cellular energy sensor. However, the role of AMPK in the metabolic effects of PPAR agonists has not been thoroughly focused. Moreover, AMPK activation by PPAR agonists seems to be independent of the receptor activation. This intriguing action of PPAR agonists may account in part for the mechanistic basis of the therapeutics in the treatment of metabolic disease. In this paper, the effects of PPAR agonists on metabolic functions were summarized with particular reference to their AMPK activity regulation.

## 1. Introduction

**Figure 1:**
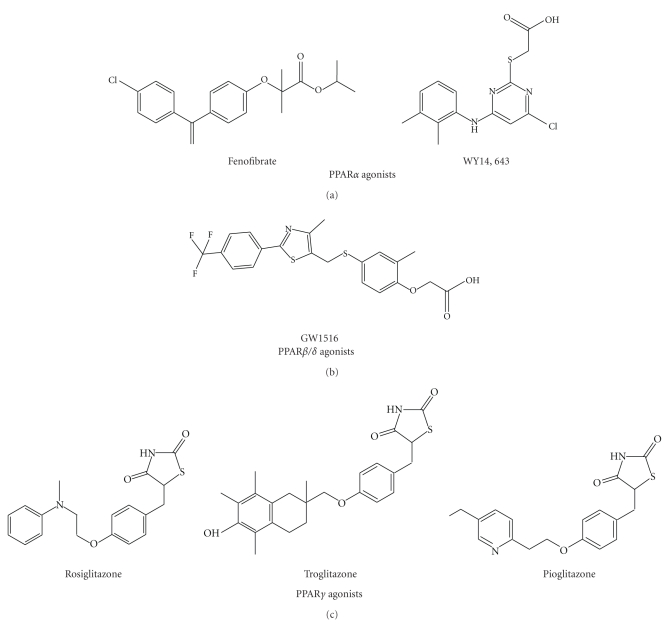
The chemical structures of PPAR activators.

The peroxisome proliferator-activated receptors (PPARs) are transcription factors that regulate diverse physiological and pathological processes including cell growth, morphogenesis, differentiation, and homeostasis [1]. PPARs are well-characterized receptors that belong to the nuclear hormone receptor superfamily: they were originally isolated as nuclear receptors that activate the proliferation of peroxisomes in the cell in 1990s [1–3]. PPARs consist of three isoforms (i.e., PPAR*α*, PPAR*β*/*δ*, and PPAR*γ*), whose tissue distributions and functional roles are distinct [4]. After the discovery, intensive studies on the biology of PPARs and their modulation by synthetic ligands have been conducted. Thus, a myriad of research have identified natural or synthetic PPAR ligands as pharmaceutical agents in the treatment of metabolic disorders [1, 4] (Figure 1). Recently, it has been recognized that PPAR agonists have physiological effects by modulating the activity of AMP-activated protein kinase (AMPK), an important cellular energy sensor [5–9]. However, this action seems to be independent of PPAR receptor activation [5, 6, 9]. These findings suggest the concept that PPAR agonists exert their effects cooperatively or synergistically with their cellular partners or associated components. This paper focuses on the effects of PPAR agonists on metabolic functions with particular reference to AMPK activity modulation.

## 2. Effects of PPAR Isoforms on Metabolic Functions

Like conventional nuclear receptors, each PPAR contains DNA-binding domain (DBD), ligand binding domain (LBD), and flexible hinge region. DBD has two zinc finger motifs whereas LBD contains 13 alpha helices and a small four-stranded beta sheet which composes a single domain [10]. Many nuclear receptors bind with retinoid X receptors (RXRs) as a modular heterodimeric partner, which also belong to members of the nuclear receptor superfamily; the RXRs possess a highly conserved central DNA-binding domain and less conserved ligand-binding domain [11]. Like other nuclear receptors, each PPAR forms a heterodimer with RXR*α* as a permissive partner, and this complex transactivates target genes for physiological activity modulations. So, RXR*α* acts as a supporting factor for strong DNA binding of PPAR. Ligand-mediated activation of the nuclear receptor homodimers or heterodimers involves their bindings to the DNA response element containing two core recognition sequences; this consensus DNA sequence present in the promoter regions of target genes is called “peroxisomal proliferator response element” (PPRE, 5′-AGGTCA-N-AGGTCA-3′) [1]. Activation of PPARs with RXR contributes to diverse physiological processes through initiation of gene transcription, the protein products of which are required for glucose/fat metabolism, inflammation, vascular physiology, and muscle performance [1–4]. Hence, activated PPARs regulate genes involved in the adaptation of cells or organs to metabolic changes.

Three PPAR isoforms have different functional roles. PPAR*α* (NR1C1), which is mainly expressed in the liver, heart, and kidney, shows high-catabolic rates of fatty acids and peroxisome-mediated activities [4]. Long-chain polyunsaturated fatty acids (especially, 20:5n-3 and 22:6n-3) and leukotriene B4 are natural ligands for PPAR*α* whereas fibrates (i.e., hypolipidemics) and WY14643 (pirinixic acid, a nonsteroidal anti-inflammatory drug) are synthetic [12]. Activated PPAR*α* promotes the expression of genes required for fatty acid and lipoprotein metabolism in mitochondria, peroxisomes, and endoplasmic reticulum [12]. Activation of PPAR*α* by agonists or fatty acids induces peroxisomal proliferation, fatty acid oxidation, and the production of ketone bodies; PPAR*α* stimulates the influx of fatty acids into the mitochondria via carnitine palmitoyltransferase 1 (CPT1) [12]. So, a deficiency of PPAR*α* shows defects in hepatic fatty acid uptake and oxidation in an animal model [13]. PPAR*α* activation increases plasma high-density lipoprotein (HDL) levels, transporting HDL particles from peripheral tissues to the liver with a decrease in plasma triglyceride (TG) level. Therefore, PPAR*α* agonists suppress dyslipidemia observed in metabolic syndrome. Moreover, PPAR*α* regulates energy balance in the body by modulating energy expenditure. Since uncoupling proteins (UCPs) contain PPREs in their gene promoter regions, PPAR*α* activators induce UCP1 in brown adipose tissue, UCP2 in liver, and UCP3 in skeletal muscle [14–16]. Recently, the role of PPAR*α* in inflammation has emerged, implying that this receptor negatively regulates inflammatory responses [12].

Although PPAR*β*/*δ* is ubiquitously expressed in most tissues, high level of PPAR*β*/*δ* (NR1C2) is observed in skeletal muscle, suggesting that it is involved in energy metabolism [17]. PPAR*β*/*δ* is engaged in membrane lipid synthesis/turnover, and cell proliferation and differentiation [17, 18]. Long-chain fatty acids and prostacyclin might serve the endogenous ligands of PPAR*β*/*δ* *.* Erucic acid has a higher affinity for PPAR*β*/*δ* than other PPAR subtypes [19]. Activated PPAR*β*/*δ* promotes fatty acid oxidation in muscle and is thought to be engaged in the adaptation of muscle to fatty acid metabolism. Like PPAR*α*, PPAR*β*/*δ* regulates a series of genes involved in fatty acid catabolism and obesity (e.g., UCP3, CPT1 and malonyl-CoA decarboxylase) [1]. Wang et al. have shown that VP16-PPAR*δ* transgenic mice with PPAR*δ* activation in adipose tissue were resistant to high-fat diet-induced and genetically predisposed obesity and hyperlipidemia. By the same token, PPAR*δ* knockout (KO) mice showed reduced energy uncoupling and were prone to obesity under high-fat diet feeding [20]. In addition, PPAR*β*/*δ* is a sensor of very low-density lipoprotein (VLDL) in macrophages, and may play a role in fat storage [21]. In skeletal muscle, enforced expression of constitutively active PPAR*β*/*δ* augmented oxidative muscle fibers and enhances running endurance [22, 23]. Consistently, PPAR*β*/*δ* overexpression helps recover insulin resistance in obesity and enhance insulin action and glucose tolerance [22, 23]. 

PPAR*γ* (NR1C3) is expressed predominantly in the adipose tissue and, to a lesser extent, in the liver. PPAR*γ* exists as three forms (*γ*1, *γ*2 and *γ*3) by alternative splicing [1]. PPAR*γ*1 is expressed in most tissues whereas PPAR*γ*2 is present predominantly in adipose tissue. PPAR*γ*3 shows high expression in macrophages, white adipose tissue, and large intestine. The results of gene KO study showed that the homozygous KO of PPAR*γ* impaired cardiac development, resulting in intrauterine death [24]. In addition, PPAR*γ* heterozygous KO mice exhibited impaired glucose homeostasis and adipocyte function, but showed increased leptin levels [25]. The PPAR*γ* activators such as free fatty acids (FFAs), eicosanoids and thiazolidinediones (TZD) induce physiological changes through target gene induction [1, 4]. PPAR*γ* activation in adipocytes sufficiently improved systemic insulin sensitization [26]. Adipose-specific PPAR*γ* activation by transgene expression and non-TZD PPAR*γ* agonist (AG035029) treatment prevented insulin resistance equivalent to TZD treatment [26]. These results suggest that fat-specific PPAR*γ* agonists may be novel candidates for diabetes. PPAR*γ* is significantly upregulated by the PPAR*γ* activators in the liver or under certain pathophysiological states (e.g., obesity) although its basal expression is rather low [27, 28]. Activation of PPAR*γ* improves insulin sensitivity in liver and muscle, decreases the intracellular lipid level in liver and muscle, and rescues insulin receptor signaling in type 2 diabetes [29]. Also, PPAR*γ* contributes to the balance between lipid influx and efflux in macrophages by upregulating target genes (e.g., fatty acid transporters and CD36) [28].

## 3. Regulatory Role of AMPK in Metabolic Functions

**Figure 2:**
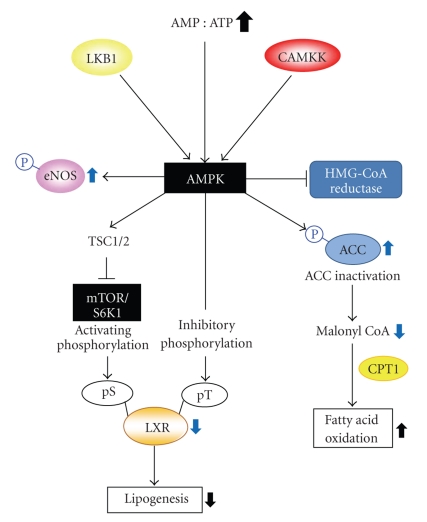
The regulatory role of AMPK in metabolic functions. CAMKK: CaM-dependent protein kinase kinase; eNOS: endothelial nitrogen oxide synthase; TSC1/2: tuberous sclerosis 1/2; mTOR: mammalian target of rapamycin; S6K1: p70 ribosomal S6 protein kinas; LXR: liver X receptor; ACC: acetyl-CoA carboxylase; CPT1: carnitine palmitoyltransferase 1.

AMPK plays a critical role in sensing and regulating energy homeostasis in cells [30]. AMPK is a serine/threonine protein kinase which physiologically responds to the change in the cellular AMP to ATP ratio. AMPK activation regulates physiological and pathological responses in diverse tissues. AMPK activation induces fatty acid oxidation in liver and heart, inhibits hepatic lipogenesis and adipocyte differentiation, and stimulates glucose uptake in muscle [30, 31]. AMPK is composed of 3 subunits (*α*, *β*, and *γ*): a catalytic subunit (*α*1 or *α*2) and two regulatory subunits (*β*1 or *β*2 and *γ*1, *γ*2 or *γ*3) [30]. AMPK activation is initiated with phosphorylation of threonine-172 in the catalytic domain of *α* subunit [31]. The *γ* subunit recognizes the AMP : ATP ratio because this has the AMP binding domain. Elevated level of AMP induces allosteric stimulation of AMPK. At present, two upstream kinases of AMPK have been discovered: LKB1 and Ca^2+^/calmodulin-dependent protein kinase kinase (CaMKK*β*) (Figure 2) [32, 33]. Transforming growth factor *β*-activated kinase-1 might be another one [34]. Both LKB1 and CaMKK directly phosphorylate thereonine-172 in AMPK*α* subunit in an AMP-independent fashion. LKB1, also known as a tumor suppressor gene, is constitutively active. The upstream kinases of LKB1 may comprise protein kinase C (PKC)-*ζ*, protein kinase A, and ribosomal S6 kinase (RSK) [35, 36]. However, several studies also suggested that there may be LKB1-independent AMPK kinase based on the findings that constitutive AMPK activity could be detected even in LKB1-deficient mice or cells (e.g., HeLa and A549 cells) [37]. In addition, the result of CAMKK activity regulation by calcium/calmodulin (Ca^2+^/CaM) indicated that AMPK might be engaged in Ca^2+^ regulation in cells. 

The important role of AMPK in glucose metabolism has been investigated in cell or animal models. Insulin regulates glucose utilization in major organs that maintain serum glucose level, such as liver and muscle. When blood glucose content is elevated, insulin secreted from the pancreatic beta cell stimulates the storage of glucose in these organs [38]. The binding of insulin to insulin receptor in the plasma membrane transmits signals that induce diverse physiological responses. Autophosphorylation of insulin receptor triggered by insulin binding leads to activation of mammalian target of rapamycin (mTOR)-p70 ribosomal S6 kinase-1 (S6K1) via phosphatidylinositol 3-kinase (PI3K)/Akt pathway. This mTOR/S6K flow is linked to AMPK. Under the condition of starvation, AMPK phosphorylates tuberous sclerosis 2 (TSC2), which inhibits mTOR/S6K1 pathway [39]: phosphorylation of TSC2 by AMPK is critical in the process of mRNA translation and cell size regulation during energy deficiency (Figure 2). Thus, AMPK activation negatively regulates the mTOR/S6K1 pathway. 

Glycogen synthase kinase-3*β* (GSK3*β*), a Ser/Thr kinase, is constitutively activated in normal state. Phosphorylation at the serine 9 residue inactivates GSK3*β*, which may promote cell survival against ischemia/reperfusion injury by blocking mitochondrial permeability transition pore opening [40]. It has been shown that resveratrol treatment inhibited GSK3*β* activity downstream of AMP-activated protein kinase (AMPK) activation, and which was responsible for mitochondrial protection [41]. AMPK induces upregulation of the p53–p21 axis, which leads to G1 cell cycle arrest. It has been reported that AICAR treatment caused cell cycle arrest in various cell types (HepG2, mouse embryonic fibroblasts, and smooth muscle cells) [42]. AICAR promotes phosphorylation of tumor suppressor p53 (Ser15 in human), subsequently leading to p21 induction. In addition, the possibility that increases in p21, p27, and p53 by AICAR inhibit proliferation of several types of cancer cells has been reported [43]. In summary, these results demonstrated that AMPK plays a pivotal role in the regulation of cell cycle and survival by affecting several downstream signals. 

AMPK activators mimic the actions of insulin in terms of gluconeogenesis, repressing glucose production [44]. Therefore, the agents that activate AMPK are beneficial in the treatment of insulin resistance and diabetes [44]. Moreover, insulin induces glucose transporter 4 (GLUT4) expression and its translocation, which facilitates glucose uptake into cells [38]. Physiologically, exercise enables GLUT4 to translocate into plasma membrane from vesicles through AMPK. The role of AMPK in exercise-induced glucose utilization is supported by the finding that treatment with aminoimidazole carboxamide ribonucleotide (AICAR), a direct AMPK activator, promoted glucose uptake, and GLUT4 translocation in skeletal muscle [45]. Consequently, 4 weeks of AICAR treatment stimulated metabolic genes and enhanced running endurance by 44% even in sedentary mice [23].

AMPK is involved in the maintenance of lipid and cholesterol homeostasis; it stimulates the *β*-oxidation of fatty acids in mitochondria for lipid utilization [44]. AMPK inhibits the activity of acetyl-CoA carboxylase (ACC) through phosphorylation (Figure 2). Under normal condition, ACC inhibits CPT1 that transports fatty acids into mitochondria and increases fatty acid oxidation. Inactivation of ACC by AMPK helps promote fatty acid utilization, leading to fat burning in liver and muscle. Liver X receptor *α* (LXR*α*) is the lipid sensor that promotes fatty acid synthesis and leads to hypertriglyceridemia. AMPK activation by metformin or dithiolethiones represses LXR*α* activity via phosphorylating threonine residue(s) of AMPK [46]. Moreover, AMPK inhibits hepatic cholesterol synthesis by inhibiting HMG-CoA reductase, a rate-controlling enzyme of the mevalonate pathway. AMPK inhibits HMG-CoA reductase activity by phosphorylation, which reduces cholesterol levels (Figure 2) [47]. Similarly, AMPK activation attenuates TG synthesis via the inhibition of LXR*α* activity in the liver, and thus results in an antisteatotic effect [46]. Of note, S6K1 activation reverses this effect of AMPK on LXR*α*-SREBP-1c pathway, as mediated by the phosphorylation of LXR*α* at serine residue. 

Vascular endothelium is usually exposed to physical stress (e.g., blood pressure or shear stress) even under normal conditions. Consequently, damaged vasculature causes blood coagulation and recruitment of immune cells. In particular, free radical stress contributes to the pathologic processes of cardiovascular diseases (e.g., atherosclerosis and coronary heart disease) [48, 49]. Apoptosis of endothelial cells by shear stress or FFAs causes injury of endothelial cell monolayer, provokes the migration of vascular smooth muscle cells (VSMCs) into the intima, and facilitates plaque formation [50]. Altered outer environment confers proliferation and migration of VSMCs, which may be stimulated by the cytokines and growth factors secreted from accumulated immune cells in the plaque. Under these conditions, uncontrolled growth of VSMCs in conjunction with endothelial cell death is critical for the development of atherosclerosis [50]. In addition, the production of reactive oxygen species (H_2_O_2_ and O_2_^−^) is amplified by the activation of NAD(P)H oxidase, peroxidase, and cyclooxygenase in these cells. AMPK may affect vascular physiology. AMPK activators including rosiglitazone and pioglitazone suppress high-glucose-induced hyperactivity of NAD(P)H oxidase in human umbilical vein endothelial cells [51, 52]. In addition, hypoxia-activated AMPK stimulates Akt that phosphorylates and activates endothelial nitrogen oxide synthase (eNOS) (Serine 1177) (Figure 2) [53, 54]. Thus, eNOS contributes to the survival and function of endothelial cells through nitric oxide (NO) production. Since AMPK responds to external stress and regulate cellular homeostasis, its activation enables endothelial cells to survive against severe stress [53, 54].

## 4. The Link between PPAR Agonists and AMPK-Dependent Metabolic Functions

### 4.1. Energy Metabolism

The metabolic disorder is a constellation of impaired glucose/lipid metabolism, hypertension, obesity, diabetes, and cardiovascular diseases [38]. Major causes of the metabolic disorder include overweight, physical inactivity, and high-carbohydrate diet that cause the disturbance of energy metabolism. A variety of metabolic diseases are highly associated with insulin resistance, as defined by the desensitization of target cells to insulin. Insulin-resistant diabetic patients are at high risk for developing hepatic diseases. Also, peripheral insulin resistance is monitored in most patients with liver cirrhosis. The major causes of insulin resistance are genetic (~50%) and environment factors including obesity (~25%), and physical fitness (~25%) [55]. Since treatment of insulin resistance has beneficial effects on diabetes, dyslipidemia, obesity, and atherosclerosis, AMPK emerges as a therapeutic target for metabolic disorders [44].

**Figure 3:**
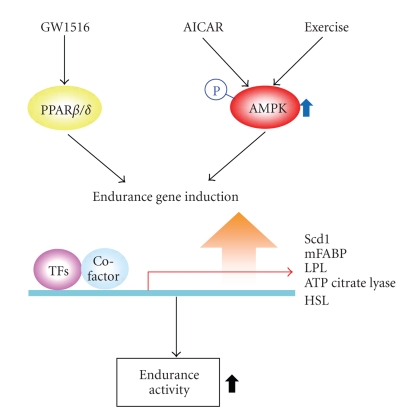
The effects of PPAR*β* */* *δ* activators on AMPK-dependent functions. AICAR: aminoimidazole carboxamide ribonucleotide; TFs: transcription factors.

Endurance exercise is the treatment recommended for patients with metabolic disease. Intriguingly, PPAR*β*/*δ* agonists serve exercise mimetics, as do AMPK activators [23] (Figure 3). Because PPAR*β*/*δ* shows high expression in skeletal muscle, treatment with PPAR*β*/*δ* agonist (GW1516) reprogrammed gene expression involved in oxidative metabolism in this tissue [23]. In addition, GW1516 administration and exercise training exert synergistic effects, as shown by the improvement of running endurance in exercise-trained mice [23]. In this study, GW1516 and AICAR synergistically increased transcription of several oxidative genes in mice quadriceps (i.e., Scd1, ATP citrate lyase, hormone sensitive lipase, muscle fatty acid binding protein, Lpl, and Pdk4). AMPK directly interacted with PPAR*β*/*δ* although it did not phosphorylate PPAR*β*/*δ*; AMPK may form a transcriptional complex with PPAR*β*/*δ*, which would strengthen the receptor activity [23]. In addition, PPAR*β*/*δ* activation by GW1516 induced SIRT1 gene transcription, which regulates body physiology and metabolism [56]. These results suggest that PPAR*β*/*δ* activates AMPK probably because SIRT1 contributes to AMPK activation. 

**Figure 4:**
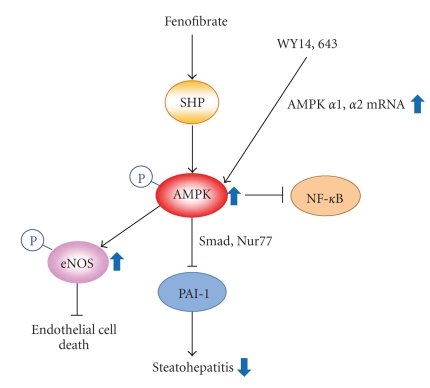
The effects of PPAR*α* activators on AMPK-dependent functions. eNOS: endothelial nitrogen oxide synthase; NF-*κ*B: nuclear factor-*κ*B; PAI-1: plasminogen activator inhibitor-1; SHP, small heterodimer partner.

PPAR*α* activators including fenofibrate and WY14643 activate AMPK signaling pathway in a receptor-independent manner (Figure 4). However, the mechanisms of these ligands on AMPK activation differ from each other. Fenofibrate induces the phosphorylation and activation of AMPK via the induction of small heterodimer partner (SHP, an orphan nuclear receptor) and its target genes [5]. On the other hand, WY14643 treatment increased the expression of AMPK*α*1 and *α*2 mRNA, leading to increase in AMPK*α* subunit phosphorylation and its enzymatic activity. However, the mechanism for this activation remains elusive [6].

**Figure 5:**
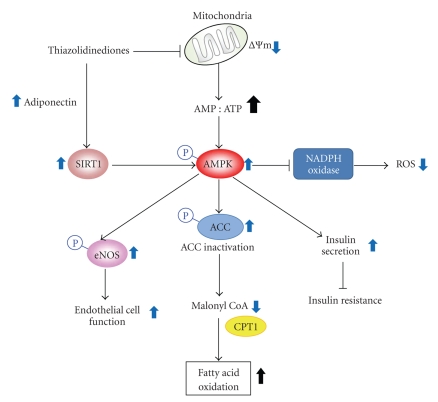
The effects of PPAR*γ* activators on AMPK-dependent functions. ACC: acetyl-CoA carboxylase; CPT1: carnitine palmitoyltransferase 1; ROS: reactive oxygen species; SIRT1: sirtuin; ΔΨm: change in mitochondria membrane potential.

PPAR*γ* agonists (i.e., TZDs) activate AMPK by phosphorylating AMPK independently of PPAR*γ* activity [7–9] (Figure 5). Troglitazone (10 mg/kg, i.p.) increased the phosphorylations of AMPK and ACC rapidly (15 min after treatment) in skeletal muscle, liver, and adipose tissue of intact rats [9]. Consistently, troglitazone caused two-fold increases in 2-deoxy-d-glucose uptake in skeletal muscle through AMPK activation. Clinically, pioglitazone also activates AMPK signaling and intensifies mitochondrial function and fat oxidation in the muscles of diabetic patients [29]. Collectively, TZDs stimulate adiponectin signals, activating AMPK, which regulates glucose metabolism and fat catabolism [29, 57].

### 4.2. Metabolic Diseases

Insulin resistance is characterized as the condition that higher level of insulin is required for normal metabolic responses because normal level of insulin fails to achieve these responses in peripheral organs. Hepatic insulin resistance causes defects in glycogen synthesis/storage and disables glucose production/release [38, 58]. Insulin resistance reduces glucose uptake in skeletal muscle, whereas it hampers the normal insulin actions and enhances hydrolysis of stored TG in fat tissue. Insulin resistance and the consequent hyperinsulinemia develop into several metabolic syndromes such as type 2 diabetes mellitus, fatty liver disease, and atherosclerosis [38, 58]. Several TZDs have been shown to recover insulin resistance via AMPK activation [59–62]. Rosiglitazone promotes AMPK-mediated insulin secretion via the phosphorylation of Kir6.2 subunit of the potassium (ATP) channel in *β*-cells [62]. However, Prentiki and his colleagues reported that pioglitazone inhibits glucose-induced insulin secretion although its antidiabetic effect depended on AMPK [60]. Therefore, the effect of pioglitazone may attribute to improve hyperinsulinemia and preserve *β*-cell function. Like pioglitazone, troglitazone restrains insulin hypersecretion at the high level of glucose and fatty acids, leading to the rescue of *β*-cells from glucolipotoxicity [59]. Recently, BLX-1002, a novel TZD with no PPAR affinity, activates AMPK in *β*-cell. BLX-1002 raises cytoplasmic Ca^2+^ and enhances glucose-induced insulin secretion at high glucose [61]. These results suggest that certain moiety(s) of TZDs is (are) responsible for AMPK activation independently of PPAR*γ* activation. In the view of glucose uptake, rosiglitazone remarkably enhanced AMPK-mediated glucose uptake and glycogen synthesis in muscle and adipose tissues [63]. Likewise, AICAR induced whole body glucose disposal (27%) and glucose infusion rate (44%), which represents improvement of insulin resistance. In cardiac muscle, PPAR*α* and PPAR*γ* activators stimulated glucose uptake via AMPK [64]. However, GW1506 (a PPAR*β* */* *δ* activator) had no effects on glucose uptake in rat L6 skeletal muscle cells [65].

Nonalcoholic fatty liver disease (NAFLD) is associated with metabolic syndrome and insulin resistance [58]. NAFLD represents the initiation step of hepatic metabolic syndrome such as steatosis, steatohepatitis, and fibrosis. Insulin resistance induced mostly by obesity may cause the development of hepatic steatosis; hyperinsulinemia augments hepatic lipogenesis of TGs and fatty acids. Several AMPK activators such as metformin and TZDs contribute to not only insulin sensitivity enhancement, but intervention of hepatic steatosis [46, 66], suggesting that signals downstream of the components activated by the drugs with different modes of action merge to the same pathway. Treatment with either rosiglitazone or pioglitazone attenuated hepatic steatosis and inflammation in patients with nonalcoholic steatohepatitis (Figure 5) [66, 67]. In addition, rosiglitazone ameliorated alcoholic fatty liver via adiponectin/SIRT1/AMPK pathway in mice [57]. Nonetheless, TZDs must be used cautiously as adjuvant therapy for nonalcoholic steatohepatitis treatment since they may provoke congestive heart failure. In recent studies, the activation of hypoxia inducible factor-1*α* (HIF1*α*)-PPAR*γ* axis promoted fatty acid uptake and glycerolipid biosynthesis genes, leading to cardiac hypertrophy [68]. In C57BL/6 mice fed a methionine-deficient and choline-deficient (MCD) diet, fenofibrate had an effect to prevent the progressive fibrosing steatohepatitis. In this model, fenofibrate induced AMPK-mediated SHP gene expression, but reduced plasminogen activator inhibitor-1 (PAI-1) mRNA and protein expression [5]. Another PPAR*α* agonist, WY14643 also ameliorated steatohepatitis along with decrease in the gene expression involved in fatty acid synthesis [5]. 

eNOS plays a role in the endothelium homeostasis through NO production [50]. It has been shown that PPAR*α* agonists including fenofibrate and WY14643 stimulate eNOS activity and NO production in human umbilical vein endothelial cells in association with AMPK activation [69, 70]. In mouse endothelial cells, AMPK activity modulation by fenofibrate contributes to inhibiting NF-*κ*B activity, implying that the agent might attenuate atherosclerosis development (Figure 4) [69]. Rosiglitazone also reduced glucose-induced oxidative stress and increases eNOS enzyme stimulation via AMPK activation [52, 71].

## 5. Concluding Remarks

PPAR agonists have diverse metabolic effects both *in vitro* and *in vivo*. The mechanisms of action include nutrients (especially glucose and lipid) metabolism and maintenance of energy homeostasis. Since AMPK is an important regulator in energy metabolism, it may be a key downstream target of PPARs, as indicated by the convergence of PPAR agonists' actions on AMPK. Overall, the effects of PPAR agonists on AMPK-mediated metabolic functions may contribute to the recovery of insulin sensitivity or treatment of metabolic syndrome (Table 1).

## Figures and Tables

**Table 1:** The effects of PPAR agonists on AMPK-dependent metabolic functions.

Isotypes	PPAR agonists	AMPK-dependent functions			
		Liver	Skeletal muscle	Adipose tissue	Vessel
PPAR*α*	Fenofibrate	Improved IR [12] NAFLD↓ [5]	—	—	eNOS↑ [69]
	WY14,643	NAFLD↓ [5]	—	—	eNOS↑ [70]

PPAR*β* */* *δ*	GW1516	—	Oxidative metabolism↑ [23] Endurance activity↑ [23] (in cooperation with AMPK)	—	—

PPAR*γ*	Rosiglitazone	Improved IR [66] NAFLD↓ [66] ASH↓ [57]	Glucose uptake↑ [63] Glycogen synthesis↑ [63]	Glucose uptake↑ [63] Glycogen synthesis↑ [63]	Oxidative stress↓ [52, 71]
	Troglitazone	Improved IR [59] NAFLD↓ [72]	Glucose uptake↑ [9]	—	—
	Pioglitazone	Improved IR [60] NAFLD↓ [67]	Fat oxidation↑ [29] Mitochondrial function↑ [29]	—	—

ASH: alcoholic steatohepatitis; IR: insulin resistance; NAFLD: nonalcoholic fatty liver disease.

## Abbreviations

ACC: Acetyl-CoA carboxylase
AICAR: Aminoimidazole carboxamide ribonucleotide
AMP: Adenosine monophosphate
AMPK: AMP-activated protein kinase
ATP: Adenosine triphosphate
CAMKK: CaM-dependent protein kinase kinase
CPT1: Carnitine palmitoyltransferase 1
DBD: DNA biding domain
eNOS: Endothelial nitrogen oxide synthase
FFA: Free fatty acid
GLUT4: Glucose transporter 4
HDL: High-density lipoprotein
KO: Knockout
LBD: Ligand binding domain
LXR: Liver X receptor
mTOR: Mammalian target of rapamycin
NAFLD: Nonalcoholic fatty liver disease
NO: Nitric oxide
PAI-1: Plasminogen activator inhibitor-1
PI3K: Phosphoinositide 3-kinase
PPAR: Peroxisome proliferator-activated receptor
PPRE: Peroxisome proliferator response element
RXR: Retinoid X receptor
SHP: Small heterodimer partner
S6K: p70 ribosomal S6 protein kinas
TG: Triglyceride
TSC2: Tuberous sclerosis 2
TZD: Thiazolidinedione
UCP: Uncoupling protein
VLDL: Very low density lipoprotein
VSMC: Vascular smooth muscle cell.
